# Supplementary material for: Sorting at embryonic boundaries requires high heterotypic interfacial tension
Source: Nat Commun. 2017 Jul 31;8:157. doi: 10.1038/s41467-017-00146-x (PMC5537356; doi:10.1038/s41467-017-00146-x)
Supplement: Supplementary file 2 — Supplementary Software 1 [file 41467_2017_146_MOESM2_ESM.zip › PottsModel/SrcPottsModel/doc/index-files/index-3.html]

C-Index


JavaScript is disabled on your browser.


Skip navigation links


- Overview
- Package
- Class
- Use
- Tree
- Deprecated
- Index
- Help

- Prev Letter
- Next Letter

- Frames
- No Frames

- All Classes

A B C D E F G H I J K L M N O P R S T U V W 


## C

cAreaUnit - Variable in class model.Cell


CartesianCoordinates - Class in model


CartesianCoordinates(double, double) - Constructor for class model.CartesianCoordinates


cDefaultConfigFile - Static variable in class model.Lattice


cEdgeUnit - Variable in class model.Cell


Cell - Class in model


Cell(Cell.CellType, int, Lattice) - Constructor for class model.Cell


Cell(int, Lattice) - Constructor for class model.Cell


Cell(Cell) - Constructor for class model.Cell
:   Copy constructor returning a copy of the passed BaseCell with identical
    member variables including a shallow copy of the pixelList.

Cell.CellType - Enum in model


Cell.Energy - Enum in model


Cell.State - Enum in model
:   Enum class representing the different states of the cell that can be
    passed as a notification message to the different class observers.

CellCoordinatesCSVStatistic - Class in engine


CellCoordinatesCSVStatistic(PottsEngine, int) - Constructor for class engine.CellCoordinatesCSVStatistic


CellDisplay - Class in gui


CellDisplay(Cell) - Constructor for class gui.CellDisplay


CellEnergyStatistics - Class in engine


CellEnergyStatistics(PottsEngine) - Constructor for class engine.CellEnergyStatistics


CellEnergyStatistics(PottsEngine, int) - Constructor for class engine.CellEnergyStatistics


CellSet - Class in model


CellSet() - Constructor for class model.CellSet


CellShapeCSVLabel - Enum in engine


CellShapeCSVStatistic - Class in engine
:   Tracks cell shape statistics (area, perimeter, center of mass) in a separate csv file for \*\*EACH CELL\*\*
    Therefore, this statistic is memory intensive and only recommended for a very limited number of MCS.

CellShapeCSVStatistic(PottsEngine, int) - Constructor for class engine.CellShapeCSVStatistic


CellStatistic - Class in engine


CellStatistic(PottsEngine, String) - Constructor for class engine.CellStatistic


CellStatistic(PottsEngine, String, int) - Constructor for class engine.CellStatistic


checkeredLattice(int, int, int, int) - Static method in class model.LatticeUtilities


checkeredLattice(int, int, int) - Static method in class model.LatticeUtilities


cMetricName - Static variable in class engine.IsoperimetricQuotientStatistic


cMetricName - Static variable in class engine.TypeSpecificAreaStatistic


cMetricName - Static variable in class engine.TypeSpecificNearestNeighborStatistic


cMetricName - Static variable in class engine.TypeSpecificPerimeterStatistic


cNotificationSource - Static variable in class engine.PottsEngine


cNotificationSource - Static variable in class engine.StatisticsManager


cNotificationSource - Static variable in class model.Cell


cNotificationSource - Static variable in class model.Lattice


ColorScale - Class in utils


ColorScale() - Constructor for class utils.ColorScale


colorScale(Color, int) - Static method in class utils.HSLColor
:   Returns a color scale from light to dark.

colorScale(Color, int, int, int) - Static method in class utils.HSLColor


CommandLineSimulation - Class in engine


CommandLineSimulation() - Constructor for class engine.CommandLineSimulation


computeEnergyLocally(Lattice, Pixel, Pixel) - Method in interface model.IEnergyFunction
:   Computes the energy of the two pixels and their neighbours.

computeEnergyLocallyIfSwapped(Lattice, Pixel, Pixel) - Method in interface model.IEnergyFunction


ConfigurationInformationPanel - Class in gui


ConfigurationInformationPanel(PottsEngine) - Constructor for class gui.ConfigurationInformationPanel


ConfigurationPanel - Class in gui


ConfigurationPanel(Constants) - Constructor for class gui.ConfigurationPanel


ConfigurationStartMenu - Class in gui


ConfigurationStartMenu() - Constructor for class gui.ConfigurationStartMenu


Console - Class in gui


Console() - Constructor for class gui.Console


Constants - Class in model
:   Reads Constants from config file

Constants() - Constructor for class model.Constants
:   Load Constants with default params (see source code for default values).

Constants(String) - Constructor for class model.Constants
:   Load Constants from custom configuration file (missing fields populated
    with default values).

Constants(String, String) - Constructor for class model.Constants
:   Load Constants from custom configuration file and give simulation a name.

Constants.Model - Enum in model


Constants.ParameterManager - Class in model
:   Manages energy information related to this simulation.

contains(Cell) - Method in class model.CellSet
:   Function used when reading in the grid.

contains(int) - Method in class model.CellSet
:   Whether the cell set contains a cell with given ID.

Coordinates - Interface in model


copyCriterion(double, double) - Static method in class engine.PottsEngine
:   Returns whether the copy from source to target pixel should be done.

copyTo(Pixel) - Method in class model.Pixel
:   Move pixel from one cell to the other and make appropriate changes.

createPaths() - Method in class model.Constants


createVectorFromCoordinates(Coordinates, Coordinates) - Static method in class model.FiniteVector


CSVStatistic<LabelEnum extends java.lang.Enum<LabelEnum>> - Class in engine


CSVStatistic(PottsEngine, int) - Constructor for class engine.CSVStatistic


CubeCoordinates(double, double, double) - Constructor for class gui.Hexagon.CubeCoordinates

A B C D E F G H I J K L M N O P R S T U V W

Skip navigation links


- Overview
- Package
- Class
- Use
- Tree
- Deprecated
- Index
- Help

- Prev Letter
- Next Letter

- Frames
- No Frames

- All Classes
